# Immobility Following Influenza

**Published:** 1895-05

**Authors:** W. Horace Hoskins

**Affiliations:** Philadelphia


### IMMOBILITY FOLLOWING INFLUENZA. RECOVERY.

BY W. HORACE HOSKINS, PHILADELPHIA.

Subject, a bay gelding, weighing 1450 pounds, five years of age; recently received from the West. Was received by writer for treatment on March 28, though had been sick for several days before.

DIAGNOSIS. Acute catarrhal influenza; temperature, 107° F.; great weakness; marked anorexia; no pulmonary symptoms beyond increased bronchial breathing; respiration and pulse only slightly increased. Was placed on a neutral fever mixture, with tincture of cinchona every four hours, and half-ounce doses of nitrate of potash and sulphate of magnesia in the drinking water, morning and evening. Temperature came down slowly from $\frac{3}{5}$ to 1 degree a day, until a temperature of 103° was reached, where it remained almost stationary for three days. Would eat only corn on the cob and but little hay; some six small ears were allowed daily. Was visited daily until April 6th, when the temperature had reached 101°, with an increased disposition to eat and assuming the recumbent position regularly at night. Was visited on the 10th and found still improving. Was ordered a sun-bath in the yard for a half hour at midday, and under this change seemed better for two days, but on the third day, after taking him to the yard with some difficulty, noticed a lack of power in the front limbs, and when ready to return him to his box-stall great difficulty ensued, occupying ten minutes to remove him less than sixty feet. On visiting him the following day found great mental depression, head held low, nose almost touching the ground, indisposed to move from one position; when compelled to move staggered and almost pitched forward; fetlocks of front limbs thrown forward and musculo-nervous tremblings of the muscles of shoulder, arm and breast; pulse full and hard; very little fecal matter eliminated the preceding twenty-four hours, but slight peristalsis on auscultation; temperature 103°. Would commence to eat when head was placed in feed-box, but would seemingly forget and cease to masticate or make any disposition of food in the mouth for two or three minutes at a time. The following day all the symptoms were more pronounced, though laxative treatment, with stimulants to the nervous system were given for twenty-four hours. On the 15th the symptoms became more alarming, and I had the animal removed to my sanitarium with ambulance. On the morning of the 16th I gave him an ear of corn, and after placing it between his nipper teeth he rested the end of the cob on the floor and apparently found comfort in thus sustaining the weight of his head for a period of three minutes. Co-ordination of the front limbs almost lost. Placed him under one-ounce doses of artificial Carlsbad salts, with dram doses of powdered rhubarb every four hours. Improvement noticeable in twenty-four hours. In four days walked fairly well, pulse became softer, appetite returned, head assumed normal position, increased action of bowels established, and temperature fell to 101°. On the 21st was exercised slightly and discharged from the sanitarium on the 25th, when he was able to trot to the halter without any visible signs of loss of co-ordination, and all the functions of the body being performed in a normal manner.

